# High dietary cation-anion difference as a biological coolant: Mechanisms of enhanced evaporative heat dissipation and comfort in tropical dairy ruminants under natural high ambient temperature

**DOI:** 10.14202/vetworld.2026.2304-2317

**Published:** 2026-06-05

**Authors:** Sumpun Thammacharoen, Sapon Semsirmboon, Nungnuch Saipin, Thomas A. Lutz, Narongsak Chaiyabutr, Thiet Nguyen

**Affiliations:** 1 Department of Physiology, Faculty of Veterinary Science, Chulalongkorn University, Bangkok 10330, Thailand; 2 Department of Anatomy, Faculty of Veterinary Science, Chulalongkorn University, Bangkok 10330, Thailand; 3 Division of Agricultural Technology, Faculty of Science, Ramkhamhaeng University, Bangkok 10240, Thailand; 4 Institute of Veterinary Physiology, Vetsuisse Faculty, University of Zurich, Zurich, Switzerland; 5 The Academy of Science, The Royal Society of Thailand, Bangkok 10300, Thailand; 6 Queen Saovabha Memorial Institute, The Thai Red Cross Society, Bangkok 10330, Thailand; 7 Faculty of Animal Sciences, College of Agriculture, Can Tho University, Can Tho 900000, Vietnam

**Keywords:** acid-base balance, dairy goats, dietary cation-anion difference, evaporative heat dissipation, heat stress, livestock welfare, thermoregulation, tropical climate

## Abstract

High ambient temperature (HTa) is a major environmental challenge affecting the welfare, physiological stability, and productivity of dairy ruminants raised in tropical and subtropical regions. Heat stress caused by HTa compromises feed intake, acid–base balance, water metabolism, thermoregulation, and milk production. This review summarizes the physiological, behavioral, and endocrinological responses of dairy goats and cows exposed to HTa conditions and highlights the potential role of high dietary cation–anion difference (hDCAD) supplementation as a nutritional strategy to mitigate heat stress. Under HTa conditions, dairy ruminants increase respiratory rate and panting to enhance evaporative heat dissipation, which may induce respiratory hypocapnia and alter electrolyte balance. The reviewed evidence demonstrates that hDCAD supplementation improves heat dissipation efficiency by increasing nocturnal water intake, expanding body water compartments, and supporting hydration status, thereby reducing rectal temperature increments during daytime heat exposure. In addition, hDCAD positively influences ruminal fermentation, nutrient digestibility, eating behavior, and dry matter intake. Long-term supplementation also promotes renal compensatory responses that help restore acid–base equilibrium. The concept of hDCAD as a “biological coolant” is proposed based on its ability to support physiological cooling mechanisms rather than solely improving feed intake or buffering capacity. This nutritional approach may improve animal comfort and productivity under tropical HTa conditions and could complement existing environmental heat mitigation strategies in dairy production systems. Further investigations in dairy goats and cows under practical farming conditions are required to strengthen the application of hDCAD as a sustainable heat stress management strategy.

## INTRODUCTION

Ruminants were domesticated approximately 8,000–10,000 years ago, and milk from dairy ruminants emerged as an important component of the human diet during the prehistoric pastoral period, approximately 5,000 BC [1, 2]. The lactation trait has been successfully selected in cattle, and dairy cows subsequently became the predominant dairy ruminant species worldwide [3]. Nevertheless, in several countries and cultures, the population of dairy goats has increased substantially, and goats have contributed significantly to total milk production [4]. In addition, dairy farming systems have progressively expanded from temperate to tropical regions to satisfy the increasing global demand for milk [5].

Ambient temperature (Ta) is an important environmental factor that negatively affects dairy ruminants. Tropical climates are generally characterized by persistently high year-round temperature-humidity index (THI) values caused by high ambient temperature (HTa) and fluctuating relative humidity (RH), which are strongly influenced by monsoon patterns [6–8]. It should be noted that this review uses Ta to represent THI. Although both Ta and RH are major determinants of THI, Ta was emphasized because daytime RH generally demonstrates an inverse relationship with Ta [11]. Furthermore, the future trend of Ta is largely influenced by global warming associated with greenhouse gas emissions and climate change [8, 9]. These climatic conditions restrict evaporative heat dissipation and activate various physiological responses in ruminants, including HTa responses and heat stress [10].

Under both natural and experimental HTa conditions, milk production in dairy cows and goats has been compromised [11, 12]. Reduced milk production in dairy cows and goats exposed to HTa has previously been reported in Thailand. Specifically, milk yield declined by approximately 16% in dairy cows and 50% in dairy goats when summer and winter periods were compared. Milk production in dairy cows decreased from 22.3 ± 1.3 kg during winter to 18.3 ± 0.7 kg during summer, whereas milk production in goats decreased from 3.2 ± 0.2 kg to 1.5 ± 0.1 kg, respectively [11–13]. However, a comparatively lower degree of milk yield reduction in dairy cows and goats under HTa conditions has also been documented [14]. Growing concerns regarding the detrimental effects of HTa on livestock welfare and production performance have encouraged researchers to develop innovative strategies capable of mitigating the adverse effects of heat stress in dairy ruminants.

Despite extensive investigations regarding the effects of HTa on dairy ruminants, previous studies have primarily focused on production losses, dry matter intake (DMI), acid-base balance, and general physiological stress responses, particularly under controlled experimental conditions. Similarly, most reports evaluating dietary cation-anion difference (DCAD) mainly emphasized ruminal buffering capacity, mineral metabolism, and milk production. However, limited information is available regarding the integrated behavioral, physiological, and renal mechanisms through which hDCAD mitigates HTa under natural tropical environments, especially in dairy goats. Furthermore, the relationships among drinking behavior, hydration status, body fluid compartment expansion, panting efficiency, evaporative heat dissipation, and renal electrolyte handling during prolonged hDCAD supplementation remain insufficiently understood. Previous studies have also rarely addressed the chronological adaptations occurring under naturally fluctuating tropical ambient temperatures, which differ considerably from climatic chamber models. Therefore, a comprehensive mechanistic framework describing hDCAD as a biological coolant strategy for dairy ruminants raised under HTa conditions is still lacking.

The present review aimed to comprehensively summarize the behavioral, physiological, endocrinological, and metabolic responses of dairy ruminants exposed to HTa conditions, with particular emphasis on tropical production systems. In addition, this review aimed to evaluate the role of hDCAD supplementation as a nutritional strategy for mitigating the adverse effects of HTa in dairy goats and cows. Specifically, the review focused on elucidating the mechanisms through which hDCAD modifies drinking and eating behavior, improves hydration status and body fluid distribution, enhances evaporative heat dissipation through panting, regulates acid-base homeostasis and renal electrolyte handling, and subsequently alleviates rectal temperature elevation under HTa conditions. Furthermore, the review proposes the novel concept of hDCAD functioning as a biological coolant and discusses its practical application as an integrated nutritional management strategy to improve animal welfare, thermal comfort, and productivity in dairy ruminants raised in tropical climates.

## REVIEW METHODOLOGY

This review was conducted using a narrative and integrative review approach to synthesize current evidence regarding the role of hDCAD as a nutritional strategy for mitigating heat stress in dairy ruminants under HTa conditions. The review focused primarily on physiological, behavioral, endocrinological, renal, and nutritional mechanisms associated with hDCAD supplementation in dairy goats and cows.

Relevant scientific literature published in English between 1980 and 2025 was retrieved from electronic databases, including PubMed, Scopus, Web of Science, ScienceDirect, and Google Scholar. The search strategy combined keywords and Boolean operators such as “dietary cation-anion difference,” “DCAD,” “high ambient temperature,” “heat stress,” “dairy goats,” “dairy cows,” “evaporative heat dissipation,” “panting,” “acid-base balance,” “electrolytes,” “water intake,” “ruminants,” and “tropical conditions.” Additional articles were identified through manual screening of reference lists from relevant publications.

Studies were considered eligible when they investigated: (i) physiological or behavioral responses of dairy ruminants to HTa or heat stress, (ii) the effects of DCAD manipulation on acid–base balance, feed intake, milk production, water metabolism, or thermoregulation, and (iii) mechanisms related to respiratory heat dissipation, renal electrolyte handling, or hydration status in ruminants. Both experimental and observational studies involving dairy goats, dairy cows, sheep, buffaloes, and related ruminant models were included when they contributed mechanistic or applied insights relevant to the review objectives.

The selection process emphasized studies conducted under natural tropical conditions because the present review aimed to reassess hDCAD as a biological coolant under realistic environmental exposure rather than exclusively controlled climatic chamber conditions. Priority was also given to studies examining diurnal and seasonal variations in HTa, drinking behavior, respiratory responses, and body fluid compartment changes.

Data extracted from the selected studies included ambient temperature conditions, THI or Ta measurements, dietary electrolyte composition, DCAD values, physiological variables (rectal temperature, respiratory rate, blood gas indices, plasma electrolytes, cortisol, and acid–base balance), behavioral responses (drinking patterns, feed intake, and meal behavior), and production outcomes such as milk yield and digestibility. The findings were subsequently organized into thematic sections addressing: (i) physiological effects of HTa and heat stress, (ii) manipulation of DCAD in dairy animals, (iii) effects of hDCAD on body hydration and evaporative heat dissipation, (iv) renal and acid–base adaptations, and (v) implications for animal welfare and milk production.

Because this article is a narrative review, no formal meta-analysis or quantitative pooled analysis was performed. Instead, the evidence was critically interpreted to develop a mechanistic framework supporting the concept of hDCAD as a biological coolant for tropical dairy ruminants.

## PHYSIOLOGICAL EFFECTS OF HTa AND HEAT STRESS

The response mechanisms of mammals to HTa conditions can chronologically be categorized into behavioral and physiological responses. The magnitude of these responses depends on the severity and duration of HTa exposure, which are influenced by the temperature difference (∆Ta) and exposure period. Acute HTa exposure is commonly established under experimental conditions in which animals are transferred from a thermoneutral Ta to a high Ta environment [15–18]. This condition differs from natural HTa exposure, where animals remain under ambient environmental conditions and Ta gradually increases and decreases throughout the day or between seasons. Natural HTa exposure can therefore be represented by comparing morning and afternoon conditions as a short-term daytime effect or comparing winter and summer periods as a long-term seasonal effect [11, 19].

Before discussing the effects of HTa in dairy ruminants, it is important to consider the current climatic conditions in tropical regions in relation to global warming. In Thailand, for example, the mean annual Ta increased by approximately 1°C over the last 100 years [8]. Current environmental observations from our group demonstrated persistent year-round HTa, in which the average monthly afternoon Ta ranged from 32.6°C to 35.2°C with fluctuating RH influenced by monsoon patterns [6]. The maximum afternoon Ta difference between winter and summer periods was approximately 4.2 ± 0.85°C. Furthermore, the reported daytime ∆Ta between morning and afternoon ranged from 5°C to 10°C [6, 11, 12]. These climatic conditions indicate three major scenarios associated with ongoing global warming: (1) short-duration Ta variation between morning and afternoon (diurnal ∆Ta), (2) gradual seasonal development of HTa between winter and summer periods (seasonal ∆Ta), and (3) sporadic HTa associated with heat waves.

Most of our information regarding HTa responses in ruminants originated from investigations based on diurnal and seasonal ∆Ta conditions. Under tropical conditions, both forms of ∆Ta coexist, and the balance between heat dissipation mechanisms and metabolic heat production plays a critical role in determining the severity of heat stress. Our findings demonstrated that dairy goats exposed to diurnal HTa conditions may experience both the first and second phases of heat stress depending on the magnitude of daytime ∆Ta (diurnal ∆Ta = 5–8°C). The first phase is mainly characterized by behavioral and physiological adaptations, whereas the second phase involves activation of the hypothalamic-pituitary axis (HPA axis), indicating stress responses in goats [10].

**Figure 1:**
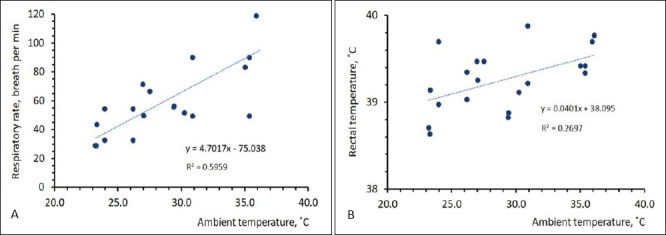
Relationship between (A) respiratory rate and (B) rectal temperature and ambient temperature (Ta). Respiratory rate (R = 0.77, p < 0.01) showed a stronger correlation with Ta than rectal temperature (R = 0.51, p < 0.05).

An increased breathing rate or panting (video clip 1; Supplementary material) is one of the earliest physiological responses during the first phase of HTa exposure and functions to enhance evaporative heat dissipation. Regardless of hydration status, increased respiratory rate represents the primary evaporative cooling mechanism in goats [15]. In goats, breathing rate closely correlates with daytime Ta fluctuations (Figure 1a), and this correlation is stronger than that observed with rectal temperature (Tr; Figure 1b). The primary physiological consequence of short-term natural HTa exposure, particularly during diurnal ∆Ta, is respiratory hypocapnia. This condition is characterized by a marked reduction in the partial pressure of carbon dioxide (PCO_2_), whereas blood bicarbonate (HCO_3_^−^) concentration and blood pH remain within normal physiological ranges without significant alterations [19–21]. Under typical diurnal ∆Ta conditions (5–8°C), PCO_2_ decreases by approximately 2 mmHg [20]. Interestingly, this natural HTa-induced respiratory hypocapnia occurred without activation of the HPA axis [19].

A greater magnitude of diurnal ∆Ta (11–25°C) has been shown to induce respiratory hypocapnia together with a tendency toward alkalosis. Under natural HTa conditions with a diurnal ∆Ta of 11–13°C, compensatory metabolic responses included reduced HCO_3_^−^ concentration without alterations in blood pH. However, respiratory alkalosis was observed only under experimental climatic chamber conditions, where ∆Ta ranged from 15°C to 25°C [16,22-25]. Comparable physiological responses have also been reported in dairy cows [26]. Moreover, HTa-induced disturbances in acid-base homeostasis affect plasma electrolyte balance. Specifically, plasma sodium (Na+) concentration and renal Na+ excretion tend to increase, whereas potassium (K+) excretion remains relatively unchanged, resulting in elevated strong ion difference values and suggesting a tendency toward alkalosis [22, 24, 25, 27].

The chloride shift phenomenon is another important physiological mechanism associated with carbon dioxide transport and plasma bicarbonate regulation. Under HTa-induced hypocapnic conditions, plasma chloride ions (Cl−) are expected to increase because of downregulation of the bicarbonate-chloride transporter [23, 28]. Nevertheless, these acid-base and electrolyte responses appear to occur chronologically during natural HTa exposure. For example, diurnal HTa conditions with ∆Ta = 5°C decreased PCO_2_ without altering plasma Cl− concentrations in dairy goats [20].

**Figure 2:**
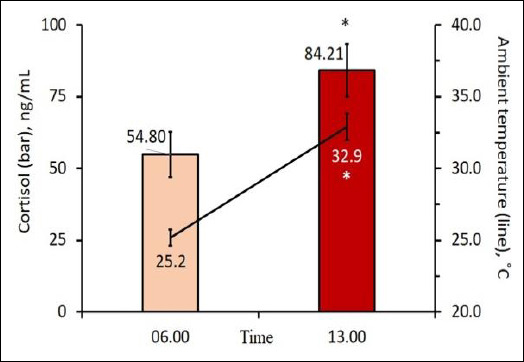
Diurnal ambient temperature (Ta) activates the hypothalamic-pituitary-adrenal axis in goats. Morning and afternoon Ta values (bars) with a ∆Ta of approximately 8°C and plasma cortisol concentrations (line) are presented. Statistical significance is indicated by an asterisk (*, p < 0.05).

When goats were transferred into climatic chambers with HTa conditions (∆Ta = 15°C), respiratory alkalosis developed together with markedly elevated plasma cortisol concentrations [16]. However, the threshold magnitude of HTa capable of activating the HPA axis under natural conditions appears more refined. Previous observations indicated that daytime ∆Ta ranging from 8°C to 10°C activated cortisol secretion in goats (Figure 2) [10, 11, 19]. In addition, blood glucose concentrations substantially increased during the transition between morning and afternoon under diurnal ∆Ta conditions of 5–8°C [19]. Similar findings, including elevated plasma glucose levels, were also observed in our previous acute heat stress model in buffaloes exposed to ∆Ta = 11°C within 4 h.

**Figure 3:**
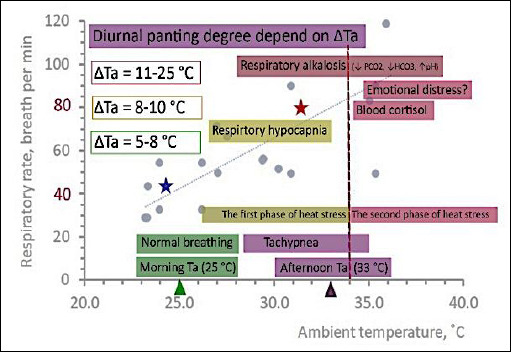
Proposed physiological responses in goats exposed to high ambient temperature (HTa) conditions. Under natural tropical conditions, the average Ta during the morning and afternoon is approximately 25°C and 33°C, respectively. During the early morning, the normal respiratory rate (blue star) was maintained at 44 ± 5 breaths/min. Increased respiratory rate progressing to tachypnea or panting (77 ± 9 breaths/min, red star) gradually developed during afternoon HTa exposure. Respiratory hypocapnia, indicated by decreased partial pressure of carbon dioxide (↓ PCO_2_), represents the primary physiological response when morning-to-afternoon Ta differences (∆Ta) range between 5°C and 8°C. Increased plasma cortisol concentrations were observed when diurnal ∆Ta exceeded 8°C. A tendency toward alkalosis, including reduced plasma bicarbonate concentration (↓ HCO_3_^−^) and increased blood pH, became evident when ∆Ta ranged from 11°C to 25°C. The first and second phases of heat stress were defined according to plasma cortisol concentrations (vertical broken line).

Taken together, breathing-induced evaporative heat dissipation appears to be the primary physiological adaptation during short-term diurnal HTa exposure. Depending on the magnitude of ∆Ta, these responses may subsequently influence acid-base homeostasis, leading to respiratory hypocapnia and eventually respiratory alkalosis (Figure 3). These physiological responses generally increase during the early morning and decline from late afternoon to evening. Importantly, our findings propose a novel chronological framework describing natural HTa responses in tropical goats. This framework distinguishes short-term respiratory hypocapnia occurring without HPA activation at ∆Ta of 5–8°C from stress-associated responses occurring at >8–10°C. These responses differ substantially from acute climatic chamber models and therefore provide a more ecologically relevant basis for developing nutritional interventions under tropical production systems. Breathing-induced evaporative heat dissipation was also observed under seasonal HTa conditions.

When winter and summer conditions in tropical environments were compared, lower daily feed intake (FI) together with panting was observed without significant differences in plasma cortisol concentrations [11]. Experimental evidence from laboratory rats demonstrated that low-magnitude HTa exposure reduced FI at thresholds lower than those required for HPA axis activation. This suggests that HTa-induced suppression of eating behavior may occur independently of, or before, systemic stress responses [29]. These findings indicate that eating behavior suppression represents an early behavioral response to HTa before activation of endocrine stress pathways. Consequently, enhanced heat dissipation through panting during short-term heat exposure and reduced FI during prolonged HTa exposure may represent the initial physiological phases of adaptation to natural HTa conditions.

**Figure 4:**
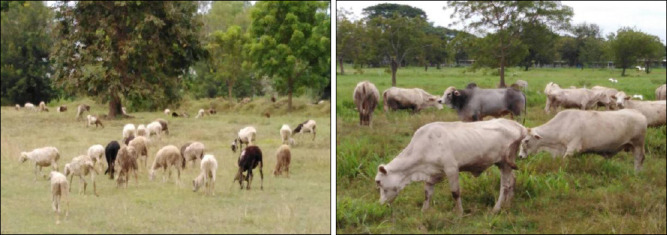
Grazing behavior under HTa conditions. (A) Afternoon grazing activity of mixed-breed ruminants near the barn represents normal behavior under HTa conditions. (B) Comparable grazing behavior was observed in zebu cattle raised in Thailand.

Our previous investigations further demonstrated that activation of the HPA axis in outdoor goats may occur when daytime ∆Ta exceeds 8–10°C (Figure 3) [10, 11, 19]. However, whether goats experience emotional distress during this second phase of heat stress remains unclear [30]. This uncertainty is supported by observations that goats and cattle continue normal grazing activities outdoors under tropical HTa conditions without displaying apparent distress behaviors during daytime periods (Figure 4 and video clip 2 [supplementary material]).

Milk yield in dairy cows and goats was compromised during both the first phase and the intermittent second phase of heat stress [6, 11–13]. The physiological mechanisms through which HTa affects milk synthesis have been proposed to involve both indirect and direct pathways associated with mammary gland function. The direct effect of HTa may alter milk synthesis through activation of heat shock protein 70 (Hsp70), whereas the indirect effect suppresses eating behavior and reduces metabolic nutrient partitioning toward the mammary gland [10, 11]. Furthermore, HTa may directly influence intracellular metabolic pathways through activation of the mTOR/HSF1/Hsp70 signaling pathway, thereby altering posttranslational processes associated with milk synthesis [10]. This phenomenon has likely intensified because of global warming and is increasingly observed in dairy animals worldwide [31–33].

Therefore, effective strategies to alleviate the adverse effects of HTa are essential for improving animal welfare and maintaining dairy productivity. Most currently available mitigation approaches primarily focus on environmental or physical modifications [14, 34]. However, our investigations demonstrated evidence supporting nutritional interventions capable of modifying body temperature homeostatic mechanisms in dairy goats. Consequently, we propose that such nutritional strategies may also be applicable to other ruminant species.

## MANIPULATING THE DCAD IN DAIRY ANIMALS

The hDCAD concept is a well-established nutritional management strategy used to improve health and productivity in dairy animals. The basic formula used to calculate DCAD is expressed as (Na+ + K+) − (Cl− + S2−) in milliequivalents [35]. In the present review, hDCAD refers to formulations containing 34–40 mEq/100 g dry matter, whereas low DCAD refers to formulations with negative DCAD values in both dairy goats and cows [20, 27, 36]. Considerable attention has been directed toward the effects of hDCAD on milk production and the effects of low DCAD on calcium homeostasis [34-35].

The present review focuses on the role of hDCAD in mitigating heat stress and improving milk production in dairy animals. Previous reports describing both positive and negative effects of hDCAD on milk yield suggest that the contribution of hDCAD to milk synthesis may depend on several external factors, including diet composition and ambient environmental conditions [27, 37–41]. Nevertheless, the beneficial effect of hDCAD on milk production under HTa conditions has partly been attributed to its influence on DMI in dairy cows and goats [35, 37, 38]. Because the direct mechanism of DCAD manipulation through cation or anion balancing involves modification of acid-base homeostasis within the gastrointestinal tract and body fluids, the effects of hDCAD on ruminal and plasma buffering capacities have been proposed as mechanisms underlying the hDCAD-induced increase in DMI [37, 38].

Based on these proposed mechanisms, the influence of hDCAD on DMI and maintenance of milk yield may involve two major pathways. First, hDCAD has been shown to improve ruminal function by supporting the ruminal buffering system and modifying fermentation characteristics, including digestibility, fermentation products, and microbial activity [38, 42–44]. Improvement of gastrointestinal tract function is considered a fundamental factor supporting dietary intake. The second pathway involves the effects of hDCAD supplementation on body fluid distribution and acid-base homeostasis, which subsequently influence DMI. The present review proposes an apparent behavioral mechanism through which hDCAD may partially support DMI under HTa conditions. Data concerning the effects of hDCAD supplementation were derived from an 8-week feeding trial in dairy goats. Detailed descriptions of the animal models, diets, and experimental procedures have been reported previously [27, 41]. The subsequent discussion suggests that the ability of hDCAD to restore core body temperature in goats exposed to HTa conditions may be more important than its direct effect on FI.

## hDCAD RESTORES BODY TEMPERATURE BY MODIFYING DRINKING PATTERNS AND BODY FLUID COMPARTMENTS AND INCREASING EVAPORATIVE HEAT DISSIPATION

**Figure 5:**
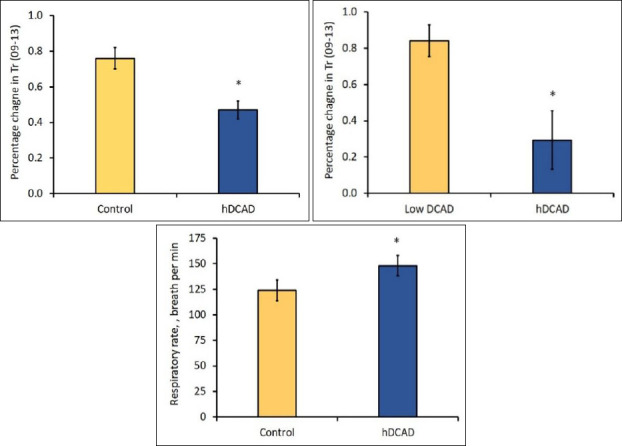
Percentage change in rectal temperature increment from morning (09:00) to afternoon (13:00) between (A) control feed and high dietary cation-anion difference (hDCAD) feed and (B) low and high DCAD feeds. (C) Effect of hDCAD supplementation on afternoon respiratory rate. Statistical significance is indicated by an asterisk (*, p < 0.05).

One of the major novel contributions of the present review is the demonstration that hDCAD functions as a biological coolant through a multifactorial synergistic mechanism. Increased nocturnal water intake expands ruminal and extracellular water reserves, which subsequently support enhanced panting efficiency during daytime HTa exposure without proportional increases in energy expenditure. This phenomenon may represent a “paradoxical tachypnea” response that has not previously been associated with DCAD supplementation. hDCAD supplementation has been demonstrated to reduce the increase in rectal temperature (Tr) during the transition from morning-to-afternoon (Figure 5a). This finding suggests a beneficial role of hDCAD supplementation for dairy ruminants raised under HTa conditions in tropical regions.

The effect of hDCAD on Tr was especially evident when goats were fed either low DCAD or hDCAD formulations (Figure 5b) [27, 45]. This response coincided with a tendency toward increased DMI [45-46]. The increase in DMI suggests that goats receiving hDCAD supplementation experienced reduced HTa-induced stress [29]. In addition, increased respiratory rate or tachypnea induced by hDCAD supplementation appears to represent an important physiological mechanism contributing to heat dissipation (Figure 5c).

Core body temperature is tightly regulated by neuroendocrine mechanisms [46]. In goats, Ta influences core temperature through diurnal fluctuations in Tr rather than seasonal changes, at least under our experimental conditions. Afternoon Tr was consistently higher than morning Tr. Importantly, despite the physiological increase in ∆Tr of <1°C during the diurnal period, plasma cortisol concentrations remained unchanged, suggesting that the HPA axis was not activated [10, 11, 19]. The percentage increase in goat Tr from early morning (09:00; 39.30 ± 0.10°C) to afternoon (13:00; 39.60 ± 0.11°C) was approximately 0.76 ± 0.06%. During hDCAD supplementation, this increment decreased to 0.47 ± 0.05% (Figures 5a and b) [27]. Similar effects of hDCAD in maintaining lower Tr under HTa conditions have also been demonstrated in sheep [48].

The proposed mechanisms through which hDCAD reduces Tr increments involve four major behavioral and physiological adaptations: increased drinking behavior, enhanced body hydration with expansion of body fluid compartments (transcellular and extracellular compartments), increased respiratory rate, and altered renal handling of potassium and chloride.

hDCAD supplementation increased daily water intake (WI) and altered drinking behavior patterns. Goats receiving hDCAD supplementation consumed more water during nighttime, whereas daytime water consumption remained comparable with that of goats receiving the control diet [27, 46]. However, several studies reported no significant changes in WI during hDCAD supplementation [41, 49]. Physiologically, thirst regulation may be activated through both pre-systemic and systemic mechanisms [50]. Ruminal osmolarity appears to play a limited role in drinking regulation [51], and increased WI during hDCAD supplementation occurred without alterations in ruminal fluid osmolarity [43]. These findings indicate that the effects of hDCAD on WI and drinking behavior are complex and may primarily involve systemic regulatory signals.

The hypothesis that eating behavior and ruminal osmotic changes act as pre-systemic signals for hDCAD-induced thirst was excluded because hDCAD supplementation did not substantially alter daytime WI or ruminal fluid osmolarity [43]. Interestingly, our findings demonstrated a tendency for increased plasma osmolarity during the afternoon in hDCAD-supplemented goats [27]. This observation suggests that plasma osmolarity may represent a systemic signal contributing to thirst drive. The increased plasma osmolarity during afternoon periods likely resulted from water loss through evaporative heat dissipation under HTa conditions. Consequently, we hypothesized that this phenomenon may stimulate nocturnal thirst and drinking behavior in hDCAD-supplemented goats. This hypothesis was partially supported by observations indicating that daytime WI measurements were limited by a ceiling effect [46]. Furthermore, subsequent investigations demonstrated that providing 1.5% saline drinking water increased WI in meat goats, whereas WI decreased or remained unchanged in dairy goats and the indigenous Bach Thao breed, respectively [52–54]. These findings indicate that the effects of hDCAD on daily WI and drinking behavior may depend on daytime HTa severity, dietary electrolyte load, and breed-specific thirst regulation mechanisms.

Consistent with the increase in WI during HTa conditions, hDCAD supplementation increased total body water without altering urine output during 7 weeks of supplementation. Analysis of body fluid compartments indicated that hDCAD-induced expansion of total body water involved both extracellular and transcellular fluid compartments [27]. Ruminants possess specialized physiological adaptations that enable maintenance of water and body fluid balance under water-deficient and HTa conditions. Because the rumen serves as a major water reservoir and mobilization compartment, it likely functions as a transcellular compartment capable of retaining additional water during hDCAD supplementation in our goat model [27]. The higher apparent water balance primarily resulted from increased water retention within these compartments together with unchanged water excretion. These findings support the hypothesis that increased daily WI during hDCAD supplementation allows the rumen to store additional water, thereby supporting breathing-associated evaporative heat dissipation and reducing heat stress during daytime periods.

Under tropical HTa conditions, breathing-mediated evaporative heat dissipation represents a primary thermoregulatory mechanism for maintaining body temperature. Increased respiratory rate to the level of panting is a normal physiological response in goats during summer HTa conditions (video clip 1; Supplementary material). hDCAD supplementation further increased respiratory rate and attenuated the increment in Tr during the transition from morning-to-afternoon (Figure 5). This increase in respiratory rate during hDCAD supplementation is unlikely to be explained solely by the alkalinizing effect of hDCAD. Because HTa is the major determinant of panting during daytime periods, and because hDCAD-supplemented goats possess greater total body water due to increased nocturnal drinking, we propose that improved hydration status acts as an important intervening factor enhancing panting efficiency.

This hypothesis is partly supported by our observation that hDCAD supplementation failed to accelerate panting when increased water drinking did not occur [40]. Furthermore, dehydration has been shown to reduce panting frequency in poultry during acute heat exposure, potentially through alterations in hypothalamic thermoregulatory set points [54, 55]. Therefore, normal or positive hydration status may represent the optimal physiological condition for maximizing panting-induced evaporative heat dissipation. Additional investigations are required to determine whether enhanced body hydration directly accelerates panting frequency under HTa conditions in goats.

## EFFECT OF hDCAD SUPPLEMENTATION ON ACID-BASE HOMEOSTASIS, RENAL HANDLING OF ELECTROLYTES, AND PARADOXICAL TACHYPNEA

During the transition of Ta from early morning-to-afternoon, HTa-induced respiratory hypocapnia has been demonstrated together with corresponding alterations in blood HCO_3_^−^ concentration and blood pH [19-20]. hDCAD supplementation shifted blood pH toward alkaline conditions without altering blood HCO_3_^−^ and PCO_2_ concentrations [41]. These blood gas characteristics were accompanied by unchanged plasma Na+, K+, and Cl− concentrations; however, there was a tendency toward increased renal K+ excretion during the first 4 weeks of supplementation, followed by increased renal excretion of both Na+ and K+ during 8 weeks of hDCAD supplementation [36].

The concept of hDCAD supplementation is generally based on the provision of alkaline feed to ruminants, which theoretically could induce some degree of metabolic alkalosis. However, our overall findings suggest that hDCAD supplementation exerts only minimal alkalinizing effects in dairy goats. The physiological responses observed during hDCAD supplementation may therefore represent a secondary phase distinct from the initial phase produced by direct bicarbonate administration into circulation. Intravenous administration of sodium bicarbonate induces classical metabolic alkalosis characterized by elevated plasma HCO_3_^−^ and PCO_2_ concentrations [57]. Interestingly, decreases in plasma K+ and Cl− concentrations together with unchanged plasma Na+ concentrations observed during bicarbonate infusion were generally consistent with our observations. Nevertheless, the physiological effects of hDCAD supplementation in goats differed substantially from those reported in acute metabolic alkalosis models in humans [58].

Furthermore, renal handling of K+ appears to play a major role in acid-base regulation in ruminants because herbivorous species rely heavily on potassium homeostasis for maintaining electrolyte balance. Consequently, the blood gas alterations observed during hDCAD supplementation are unlikely to represent the primary factor regulating respiratory rate. We therefore propose that the increased breathing rate observed during hDCAD supplementation under HTa conditions represents a paradoxical physiological phenomenon associated with alkaline loading. Under combined hDCAD supplementation and HTa conditions, enhanced hydration status resulting from increased total body water may stimulate the respiratory center and improve panting efficiency in goats as a ruminant model [55, 56].

Accordingly, paradoxical panting during hDCAD supplementation under HTa conditions may require the simultaneous interaction of three major factors: HTa exposure, altered drinking behavior, and positive hydration status. If this physiological phenomenon is common among ruminants, hDCAD supplementation may represent an advantageous nutritional strategy for goats and dairy cows raised under tropical conditions.

## hDCAD SUPPLEMENTATION INCREASES EATING BEHAVIOR UNDER HTa CONDITIONS

An important knowledge gap regarding hDCAD supplementation should be addressed before this nutritional strategy can be fully integrated into the management of heat-stressed dairy goats and cows during summer periods. As previously discussed, if hDCAD functions primarily as an alkaline dietary formulation, it is necessary to determine whether this strategy is physiologically compatible with normal eating behavior in ruminants. This question should be evaluated from both behavioral and physiological perspectives.

Numerous studies have demonstrated that hDCAD formulations increase DMI [35, 37, 38]. In addition, our findings demonstrated that hDCAD supplementation increased DMI through modification of meal patterns [46]. Goats supplemented with hDCAD exhibited larger meal sizes and longer meal durations. Meal pattern analysis is widely recognized as an important approach in the neuroscience of eating behavior because it partially reflects the hedonic characteristics of food, and this concept appears to be applicable to goats as well [59, 60].

**Figure 6:**
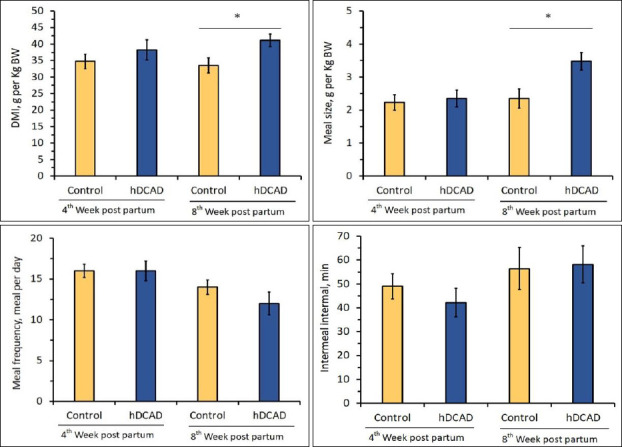
Eating patterns in lactating goats fed control or high dietary cation-anion difference (hDCAD) diets. All goats received the control diet during the first 2 weeks postpartum. Control or hDCAD diets were subsequently introduced at 2 weeks postpartum, and eating patterns were evaluated at weeks 4 and 8 postpartum. (A) DMI was higher in the hDCAD group at week 8, corresponding with increased (B) meal size. hDCAD supplementation had no effect on (C) meal frequency or (D) intermeal interval. Statistical significance is indicated by an asterisk (*).

Our current behavioral findings suggest that the effects of hDCAD on DMI may involve a time-dependent mechanism capable of modifying taste perception. This hypothesis is supported by the observation that hDCAD supplementation altered meal patterns at week 8 but not at week 4 (Figure 6). These findings indicate that hDCAD increased DMI primarily through mechanisms associated with post-absorptive signals rather than pre-absorptive signals or palatability effects [46]. Because food palatability and preference may be influenced by memory and learning processes [61], post-absorptive physiological responses enhancing taste perception may represent an important mechanism underlying hDCAD supplementation.

This hypothetical mechanism is particularly interesting and warrants further investigation under conditions where external or artificial influences are minimized [59]. In addition to the mechanisms explaining the effects of hDCAD on FI, increased DMI suggests that ruminants may exhibit altered food selection behavior that could improve animal well-being [62]. Therefore, implementation of hDCAD formulations in ruminant diets under HTa conditions during tropical summer periods may contribute to mitigation of HTa effects and improvement of animal welfare.

## CONCLUSION

HTa remains a major environmental challenge affecting dairy ruminant welfare, physiological homeostasis, and milk production, particularly under tropical production systems. The present review demonstrated that dairy goats and cows exposed to HTa exhibit coordinated behavioral, physiological, endocrinological, and metabolic adaptations, including panting, altered drinking and eating behavior, respiratory hypocapnia, acid-base adjustments, and reduced milk yield. The review further highlighted that the severity of these responses depends on the magnitude and duration of temperature fluctuations under both diurnal and seasonal ∆Ta conditions. Importantly, the findings support the concept that hDCAD supplementation can function as a biological coolant by promoting nocturnal drinking behavior, expanding body water compartments, improving hydration status, enhancing panting efficiency, and attenuating Tr increments under HTa conditions.

The review also demonstrated that hDCAD supplementation influences acid-base homeostasis and renal electrolyte handling without inducing severe metabolic alkalosis. Increased respiratory rate during hDCAD supplementation appeared to be associated with improved hydration-mediated evaporative heat dissipation rather than direct alkalinizing effects. In addition, hDCAD supplementation modified meal patterns and increased DMI, suggesting beneficial effects on feeding behavior and animal comfort during heat exposure. Collectively, these findings indicate that hDCAD supplementation may represent an integrated nutritional strategy capable of improving thermoregulation, maintaining physiological stability, and supporting milk production in dairy ruminants raised under tropical HTa conditions.

One of the major strengths of the present review is the integration of behavioral, physiological, endocrinological, and renal adaptive mechanisms into a unified framework describing hDCAD as a biological coolant. Unlike many previous investigations performed under controlled climatic chamber conditions, this review primarily synthesized findings obtained under natural tropical HTa environments, thereby providing greater ecological and practical relevance for dairy production systems in tropical regions. Furthermore, the review introduced the novel concept of paradoxical tachypnea associated with enhanced hydration status and evaporative heat dissipation during hDCAD supplementation.

Nevertheless, several limitations should be acknowledged. Most mechanistic evidence summarized in this review originated from studies conducted in dairy goats, and comparatively limited data are available for dairy cows and other ruminant species. In addition, several proposed mechanisms, particularly those involving hydration-mediated respiratory regulation, thirst signaling pathways, meal pattern modulation, and intracellular thermoregulatory responses, remain hypothetical and require direct experimental confirmation. Variations among breeds, environmental conditions, dietary formulations, and management systems may also influence the consistency of physiological responses to hDCAD supplementation.

Future studies should therefore focus on validating the biological coolant concept of hDCAD across different ruminant species, production stages, and climatic conditions. Long-term investigations evaluating milk production efficiency, reproductive performance, immune responses, metabolic adaptations, and animal welfare under practical farm conditions are also required. Moreover, further mechanistic studies should investigate the interactions among hydration status, respiratory control centers, renal electrolyte regulation, ruminal water dynamics, and molecular pathways associated with thermoregulation and milk synthesis. The integration of hDCAD supplementation with physical cooling systems and precision nutritional management strategies should additionally be explored to optimize heat stress mitigation under tropical dairy production systems.

In conclusion, the present review provides evidence that hDCAD supplementation has substantial potential as a nutritional strategy for alleviating the detrimental effects of HTa in dairy ruminants. By improving hydration status, enhancing evaporative heat dissipation, supporting eating behavior, and stabilizing physiological homeostasis, hDCAD may improve animal welfare and productivity under tropical heat stress conditions. The biological coolant concept proposed in this review provides a promising foundation for future nutritional and management innovations aimed at improving the sustainability and resilience of dairy production systems in regions increasingly affected by global warming.

## DATA AVAILABILITY

The supplementary data including video clips can be made available from the corresponding author upon a request.

## AUTHORS’ CONTRIBUTIONS

ST and TN: Conceptualization. ST: Writing – original draft. ST, TN, TL, and NC: Writing – review & editing. ST: Funding acquisition. SS, NS, and TN: Data curation. TL and NC: Supervision. All authors have read and approved the final manuscript.
